# Perspectives on public health interventions in the management of the COVID-19 pandemic in Thailand

**DOI:** 10.12688/wellcomeopenres.16293.2

**Published:** 2020-12-02

**Authors:** Wirichada Pan-ngum, Tassawan Poomchaichote, Pimnara Peerawaranun, Natinee Kulpijit, Anne Osterrieder, Naomi Waithira, Mavuto Mukaka, Bhensri Naemiratch, Rita Chanviriyavuth, Supa-at Asarath, Supanat Ruangkajorn, Noppadon Kannika, Phaik Yeong Cheah

**Affiliations:** 1 Mahidol-Oxford Tropical Medicine Research Unit, Faculty of Tropical Medicine, Mahidol University, Bangkok, 10400, Thailand; 2 Department of Tropical Hygiene, Faculty of Tropical Medicine, Mahidol University, Bangkok, 10400, Thailand; 3 Centre for Tropical Medicine and Global Health, Nuffield Department of Medicine, University of Oxford, Oxford, UK; 4 SUPER POLL, Research Institute for Community Happiness and Leadership, Mahidol University, Bangkok, 10400, Thailand; 5 UCSI Poll Research Centre, Faculty of Business and Information Science, UCSI University, Kuala Lumpur, Malaysia; 6 The Ethox Centre, Nuffield Department of Population Health, University of Oxford, Oxford, UK

**Keywords:** social, ethical, behavioural, COVID-19, pandemic, social distancing, quarantine, isolation, Thailand, survey, public health

## Abstract

**Background: **Any government needs to react quickly to a pandemic and make decisions on healthcare interventions locally and internationally with little information regarding the perceptions of people and the reactions they may receive during the implementation of restrictions.

**Methods**: We report an anonymous online survey in Thailand conducted in May 2020 to assess public perceptions of three interventions in the Thai context: isolation, quarantine and social distancing. A total of 1,020 participants, of whom 52% were women, responded to the survey.

**Results**: Loss of income was the main concern among respondents (>80% for all provinces in Thailand). Traditional media and social media were important channels for communication during the pandemic. A total of 92% of respondents reported that they changed their social behaviour even before the implementation of government policy with 94% reporting they performed social distancing, 97% reported using personal protective equipment such as masks and 95% reported using sanitizer products.

**Conclusions**: This study showed a high level of compliance from individuals with government enforced or voluntarily controls such as quarantine, isolation and social distancing in Thailand. The findings from this study can be used to inform future government measures to control the pandemic and to shape communication strategies.

## Introduction

There is a lack of data on the social, ethical and behavioural aspects of public health interventions used globally to control the coronavirus disease 2019 (COVID-19) pandemic especially from Southeast Asia. This information is important for policy makers to inform future plans to deal with the situation that is changing quickly. This paper reports the findings of a survey conducted in Thailand during the recovery period of first wave of COVID-19, i.e. the entire month of May 2020. We sought to understand the perceptions of the people on the disease and public health interventions to curb the pandemic. These findings could be useful when planning and making decisions about subsequent waves of the COVID-19 pandemic or future outbreaks. This study is a part of an ongoing multinational, mixed-methods research involving Malaysia, Thailand, Italy, Slovenia, and United Kingdom (UK) ^1^.

### COVID-19 situation between January and May 2020 in Thailand

An outbreak of COVID-19 started in December 2019 when the health authorities in China reported the first case of a novel coronavirus (severe acute respiratory syndrome coronavirus (SARS-CoV-2)) in Wuhan city of Hubei province in China. Since then, a number of confirmed cases were subsequently reported across the globe. On March 11, 2020, the World Health Organisation (WHO) declared COVID-19 a pandemic. Numbers of confirmed cases have rapidly been growing, and deaths were observed with a mortality rate of 4.6%. According to WHO, as of September 25, 2020, the confirmed cases around the globe reached 32 million cases with 979,212 deaths. The top three regions that reported the highest number of confirmed cases are the Americas (16m), South-East Asia (6.5m), and Europe (5.5m) ^2^.

Since January 3, 2020, Thailand started to implement a surveillance protocol by fever screening of travelers arriving from Wuhan, at the Suvarnabhumi, Don Mueang, Phuket, and Chiang Mai international airports. On January 13, 2020, a person traveling from Wuhan to Thailand tested positive for COVID-19, which was confirmed to be the first case in Thailand and also the first case outside China. On the same day, Thailand identified one more confirmed case, a 74-year-old female Chinese tourist. By the end of January, there were 19 confirmed cases in Thailand. All cases were travelers from abroad, except a Thai taxi driver, which was the first case in Thailand with no recent history of travel to China ^3^.

The turning point for Thailand’s COVID-19 situation was on March 12 and 13, 2020, when two big clusters of the disease were reported, one from a nightlife spot and one from a boxing stadium in Bangkok. The number of cases in Thailand rose rapidly after those two clusters were found. More than 100 cases were confirmed by the end of that week ^4^.

On March 21, 2020, the Governors of Bangkok, five neighboring provinces and Chiang Mai imposed urgent measures to ensure social distancing, including closing a range of retail businesses. Because of these urgent measures, workers from retail businesses in Bangkok traveled back to their hometowns. This increased the number of confirmed cases in provinces outside of Bangkok from 59 cases on March 19 to 236 cases on March 22. On March 26, 2020, the National Emergency Decree was issued. This decree authorizes government agencies to effect or enforce specific actions necessary to reduce transmission of the virus and bring the epidemic under control. The initial restrictions included prohibiting travelers from entering the Kingdom of Thailand except for Thai citizens, shippers, diplomats or representatives of international bodies who have to work in Thailand. The public was requested to remain inside their homes and to strictly limit all social contacts. On April 3, 2020, the government announced a nationwide curfew. All residents were instructed to remain inside their homes between the hours of 10 pm to 4 am. The government requested that everyone wore a cloth mask when outside their home. After April 9, the number of cases decreased to below 100 cases per day. A month later, on May 3, the government approved the first phase of relaxed measures. Since then, the other phases of relaxed measures have followed. As of July 1, the government approved the fifth phase of relaxed measures but still extended the enforcement of the emergency decree. Thai schools were allowed to reopen, and some high-risk businesses were allowed to resume their operations but under strict precautionary measures, including pubs, bars, karaoke bars, massage parlors, bouncy castles, ball play areas for children, bull fighting, cock fighting, fish fighting and similar activities. To accommodate journeys across provinces, all public transport (buses, vans, trains, ferries, airplanes) had to provide breaks during the journey, spacing between seats and must limit the number of passengers. On August 13, the cabinet approved the resumption of another three key activities back to normal operations. All educational institutions and schools were allowed to open with their regular schedule, public transport were allowed to resume normal service in terms of passenger numbers, and the public was allowed to join or watch any outdoor sport activities, although numbers were limited.

Up to September 25, the Ministry of Health, Thailand reported that there had been no new locally transmitted cases in the country. The only positive cases were those returning residents from different countries. They were quarantined in the government-provided facilities ^5^.

## Methods

### Study design

We conducted an online anonymous survey in Thailand of people’s opinions and perspective on the public health intervention implementation in Thailand in response to the COVID-19 pandemic. Full details of the wider study protocol of which this is a part of have been reported previously ^1^. Briefly, the “Social, ethical and behavioural aspects of COVID-19 (SEBCOV)” study consisted of an online survey and qualitative interviews in Southeast Asia (Thailand and Malaysia) and Europe (United Kingdom, Italy and Slovenia).

The survey was developed in English by the SEBCOV study team, and then translated to Thai and consisted of five domains ( *Extended* data ^6^): demographics (7 questions); income, occupation status and the economic impacts of COVID-19 and government restrictions (8 questions); COVID-19 communication that respondents had received, what they would prefer, what information had been perceived as unclear or confusing, and the occurrence of ‘fake news’(5 questions); self-reported level of understanding of COVID-19 and related restrictions, level of acceptance of these restrictions and behaviour changes, concerns relating to restrictions, and coping strategies (14 questions). The 5-point Likert-scale, ranged from strongly agree to strongly disagree, was used for opinions on statements reflecting the opinions on the government restrictions. The survey was set up using the JISC Online surveys’ platform ^7^.

The survey questions were pilot-tested with 25 people at the five participating countries prior to rollout, and revised to improve their clarity. In addition to pilot testing, selected questions were tested using an adapted cognitive testing technique using the “thinking out loud” approach ^8^, with the Bangkok Health Research Ethics Interest Group, a public involvement group set up by the Mahidol Oxford Tropical Medicine Research Unit (MORU) in August 2019. The members gave feedback on the content and phrasing of the questions, as well as on the study in general.

### Study participants and recruitment

Recruitment of respondents was done via snowball sampling using existing professional and personal contacts through email, social media and recruitment posters. Our survey was not designed to be nationally representative, but sought to compare population segments, e.g. men versus women; younger versus older people; those with higher versus lower levels of education. However, we made every effort to have geographical representation within Thailand. A polling company, SUPER POLL was engaged to help with survey dissemination. Stratification by area was applied to guarantee that respondents from all regions across the country were included in the survey: north, central, northeast or “Isan”, south, and Bangkok areas.

A total of 1,020 participants responded to the online survey in the month of May 2020. Informed consent was obtained from the participants online prior to starting the survey. Inclusion criteria were adults (18 years old and above) residing in Thailand who were able to use a computer or a smart phone. Exclusion criteria were those individuals who were illiterate since the data collection was online and the survey was self-administered.

### Sample size

A sample of 1,020 individuals responded to the survey. This exceeded the recommended rule of thumb sample size for a mixed methods study (between 40 and 200 respondents per study are recommended) ^9^ and also exceeded the planned sample size of 600, hence higher precision. Taking the rule of thumb into account for mixed methods as explained above, a target sample size of 600 minimum number of respondents was planned. The planned 600 respondents were adequate to estimate any prevalence of a response assuming a 50% prevalence rate, estimating with 95% confidence and with a precision of 4%. The 50% prevalence is the standard assumption for sample size calculations when the true prevalence is not available in literature because this gives the highest sample size for a binomial distribution for a desired level of precision. Thus, the number of individuals in this study i.e. 1,020 are adequate to estimate any prevalence of a response with approximately 3% margin of error and 95% confidence level.

### Data collection

Data were collected from 1 ^st^ May 2020 to 31 ^st^ May 2020 ( *Underlying data* ^10^). This represents the period in which the number of reported cases in the country was decreasing after the extensive restrictions by the government under the state of emergency announced at the end of April 2020.

### Ethical approval

Ethical approval for this study was received from the Ethics Committee of the Faculty of Tropical Medicine, Mahidol University, Bangkok, Thailand (TMEC 20-016) and Oxford Tropical Research Ethics Committee (OxTREC reference number 520-20). Informed consent was provided by participants online prior to completing the survey.

### Statistical analyses

The quantitative data was analysed using Stata 15.0 software. Frequency counts and percentages were used to summarise categorical data. Median and interquartile range (IQR) were used to describe the continuous data. Associations between categorical variables were assessed using the Chi squared test or Fisher’s exact tests as appropriate. A Z-test for trend has been used to assess association been binary and ordinal categorical variables. Tests of significance will be performed at 5% significance level.

## Results

### Characteristics of survey respondents

Table 1 shows the key characteristics of our respondents by region. The responses were well distributed geographically, with the smallest number of responses coming from the Eastern and Western regions. The breakdown of respondents by region is as follows: Central (28%), Northeastern (27%), Southern (19%), Northern (19%) and Eastern/Western (7%). Overall, there was a ratio of 52:47:1 female:male:other/prefer not to say respondents. The majority of the respondents were aged between 35 and 54 years old in all regions, accounting for 59% of the total respondents. Respondents from the North and Northeast regions had a relatively lower level of education compared to the rest of the country. The survey consisted of approximately 80% general population and 20% healthcare workers (HCWs). In general, HCWs were defined as people who reported working full time in the health sector (5%), while local HCWs are people who work as health volunteer staff (13%), rather than full-time. Healthcare workers have been given more in-depth health education compared to the general public and are expected to provide basic health information to local residents, and coordinate doctors’ visits during the pandemic. Finally, the testing of COVID-19 was highest in the Southern region. 

**Table 1.** Demographics of online survey participants in the COVID-19 study, Thailand.

Regions	Northern	Northeastern	Central	Southern	Eastern/Western	Total
	N=191 (%)	N=277 (%)	N=286 (%)	N=194 (%)	N=72 (%)	N=1,020 (%)
**Gender**						
Female	98 (51)	116 (42)	189 (66)	89 (46)	41 (57)	533 (52)
Male	93 (49)	161 (58)	94 (33)	104 (54)	29 (40)	481 (47)
Other/Prefer not to say	0 (0)	0 (0)	3 (1)	1 (1)	2 (3)	6 (1)
**Age (years)**						
18–24	2 (1)	8 (3)	32 (11)	13 (7)	3 (4)	58 (6)
25–34	16 (8)	13 (5)	58 (20)	10 (5)	10 (14)	107 (10)
35–44	51 (27)	96 (35)	82 (29)	36 (19)	18 (25)	283 (28)
45–54	65 (34)	92 (33)	59 (21)	88 (45)	17 (24)	321 (31)
55–64	49 (26)	48 (17)	37 (13)	37 (19)	18 (25)	189 (19)
65–84	8 (4)	20 (7)	18 (6)	10 (5)	6 (8)	62 (6)
**Education level**						
Primary school or lower	34 (18)	65 (23)	11 (4)	52 (27)	11 (15)	173 (17)
Secondary/High school/Vocational school	112 (59)	169 (61)	60 (21)	73 (38)	21 (29)	435 (43)
Bachelor degree or higher	45 (24)	43 (16)	215 (75)	69 (36)	40 (56)	412 (40)
**Living arrangements**						
Living alone	12 (6)	15 (5)	71 (25)	10 (5)	8 (11)	116 (11)
Living with partner	27 (14)	23 (8)	38 (13)	25 (13)	13 (18)	126 (12)
Living with partner and children/ others	152 (80)	239 (86)	177 (62)	159 (82)	51 (71)	778 (76)
**Household size, median (IQR)**	4 (3, 4)	4 (3, 5)	4 (2, 5)	4 (3, 5)	3 (2, 4)	4 (3, 5)
**Having the following groups in** **household**						
Children (below 18 years)	92 (48)	185 (67)	72 (25)	97 (50)	14 (19)	460 (45)
Persons aged 70 or older	34 (18)	141 (51)	68 (24)	39 (20)	11 (15)	293 (29)
Pregnant woman	2 (1)	17 (6)	7 (2)	3 (2)	1 (1)	30 (3)
People with serious health conditions	8 (4)	27 (10)	14 (5)	13 (7)	0 (0)	62 (6)
**Being a healthcare provider/** **worker**						
HCW	55 (29)	24 (9)	42 (15)	57 (29)	13 (18)	1919)
- General HCW	15 (8)	13 (5)	0 (0)	25 (13)	2 (3)	55 5)
- Local HCW (Agricultural, forestry and fishery workers)	40 (21)	11 (4)	42 (15)	32 (16)	11 (15)	136 (13)
Non-HCW ^1^	136 (71)	253 (91)	244 (85)	137 (71)	59 (82)	829 (81)
**Type of income**						
Fixed income	79 (41)	121 (44)	160 (56)	31 (16)	23 (32)	414 (41)
Unfixed income	112 (59)	156 (56)	126 (44)	163 (84)	49 (68)	606 (59)
**Occupation**						
Agricultural, forestry and fishery workers	96 (50)	118 (43)	4 (1)	94 (48)	35 (49)	347 (34)
Others	95 (50)	159 (57)	282 (99)	100 (52)	37 (51)	673 (66)
**Tested for COVID-19**	17 (9)	16 (6)	11 (4)	51 (26)	1 (1)	96 (9)

^1^ Included respondents who were not working

### Impacts of COVID-19

Table 2 summarises the economic impacts of COVID-19 and related government interventions. A total of 87% of respondents were working (paid and unpaid work) before the COVID-19 pandemic. Of those who were working, over 80% of them lost some earnings due to the pandemic. Almost 50% had their work hours reduced and experienced temporary closure of their workplace. Approximately 14% had to isolate themselves due to exposure and about 20% had to stop working during the pandemic. A switch to a “new normal” of working from home was reported by 18% of respondents. Among those who have experience working from home, 45% found it convenient and voted to continue working this way even when the pandemic is over. This was especially true among respondents in the Central region.

**Table 2.** Economic impacts of COVID-19 in each region.

	Northern	Northeastern	Central	Southern	Eastern/Western	Total
	N=191 (%)	N=277 (%)	N=286 (%)	N=194 (%)	N=72 (%)	N=1,020 (%)
**Work status (yes/no) before** **COVID-19?**	165 (86)	246 (89)	229 (80)	183 (94)	65 (90)	888 (87)
**Any inconvenience caused by** **COVID-19**						
Loss of earnings	N=163 (%) 143 (88)	N=245 (%) 222 (91)	N=227 (%) 138 (61)	N=182 (%) 168 (92)	N=65 (%) 50 (77)	N=882 (%) 721 (82)
Reduction of working hours	N=164 (%) 79 (48)	N=234 (%) 145 (62)	N=218 (%) 90 (41)	N=178 (%) 45 (25)	N=64 (%) 39 (61)	N=858 (%) 398 (46)
Closure of workplace (temporarily or indefinitely)	N=164 (%) 49 (30)	N=223 (%) 134 (60)	N=222 (%) 85 (38)	N=179 (%) 57 (32)	N=64 (%) 40 (63)	N=852 (%) 365 (43)
Heavier charge of work due to the emergency	N=163 (%) 37 (23)	N=241 (%) 87 (36)	N=221 (%) 55 (25)	N=179 (%) 42 (23)	N=64 (%) 42 (66)	N=868 (%) 263 (30)
Loss of job	N=163 (%) 47 (29)	N=221(%) 52 (24)	N=215 (%) 30 (14)	N=177 (%) 54 (31)	N=64 (%) 10 (16)	N=840 (%) 193 (23)
Temporarily isolated due to exposure	N=162 (%) 28 (17)	N=225 (%) 25 (11)	N=216 (%) 21 (10)	N=178 (%) 13 (7)	N=64 (%) 33 (52)	N=845 (%) 120 (14)
**Work during COVID-19**						
No	23 (14)	75 (30)	31 (14)	53 (29)	7 (11)	189 (21)
Yes, implementing smart- working/work from home	25 (15)	26 (11)	92 (40)	8 (4)	7 (11)	158 (18)
Yes, working as usual	117 (71)	145 (59)	106 (46)	122 (67)	51 (78)	541 (61)
**Prefer continuing smart-** **working/work from home** **after COVID-19**	N=25 (%)	N=26 (%)	N=92 (%)	N=8 (%)	N=7 (%)	N=158 (%)
Don't know	1 (4)	13 (50)	10 (11)	2 (25)	1 (14)	27 (17)
No	13 (52)	7 (27)	34 (37)	3 (38)	3 (43)	60 (38)
Yes	11 (44)	6 (23)	48 (52)	3 (38)	3 (43)	71 (45)

In Table 3, the majority of the survey participants reported that they lived with an extended family including a partner and children or relatives (76%). The highest concern among people who live with others was financial (over 80%). Those who live with an extended family were also concerned about their increased responsibilities in caring for others, and health issues related to the pandemic. On the contrary, those who live alone tended to be concerned about the impact on their social life, their mental health and wellbeing (64%).

**Table 3.** Self-perceptions and concerns on the pandemic stratified by living arrangements.

	Living alone	Living only with partner/non- relatives	Living with partner and children/relatives	Total	P-value
	N=116 (%)	N=126 (%)	N=778 (%)	N=1,020 (%)	
**Concerns when no physical contact/not** **allowed to go out/allowed to go out only** **for essential needs**					
Financial (e.g. loss of income, loss of job)	N=114 (%) 71 (62)	N=124 (%) 103 (83)	N=773 (%) 659 (85)	N=1,011 (%) 833 (82)	<0.001
Physical health (e.g. not being able to attend doctor appointments, medication supply for illnesses, lack of exercise)	N=115 (%) 79 (69)	N=124 (%) 79 (64)	N=765 (%) 541 (71)	N=1,004 (%) 699 (70)	0.28
Caring responsibilities (e.g. childcare, caring for elderly parents, not having access to care)	N=114 (%) 70 (61)	N=124 (%) 75 (60)	N=762 (%) 527 (69)	N=1,000 (%) 672 (67)	0.061
Mental health and wellbeing (e.g. boredom, loneliness, anxiety, depression)	N=112 (%) 72 (64)	N=124 (%) 66 (53)	N=740 (%) 446 (60)	N=976 (%) 584 (60)	0.20
Social (e.g. not being able to see friends or attend social or family events)	N=112 (%) 72 (64)	N=124 (%) 70 (56)	N=753 (%) 430 (57)	N=989 (%) 572 (58)	0.34
Infrastructure (e.g. access to transport, network services, internet access)	N=111 (%) 53 (48)	N=123 (%) 62 (50)	N=726 (%) 370 (51)	N=960 (%) 485 (51)	0.82
Living arrangements (e.g. not enough living space, passing on illness to family members, domestic abuse)	N=111 (%) 49 (44)	N=123 (%) 57 (46)	N=734 (%) 382 (52)	N=968 (%) 488 (50)	0.19
Religious and spiritual (e.g. not being able to go to church, mosque, temple etc.)	N=110 (%) 46 (42)	N=123 (%) 61 (50)	N=753 (%) 349 (46)	N=986 (%) 456 (46)	0.49
Professional/career progression	N=111 (%) 58 (52)	N=122 (%) 66 (54)	N=729 (%) 313 (43)	N=962 (%) 437 (45)	0.022
Sports (e.g. participating in competitive or professional sports activities)	N=112 (%) 58 (52)	N=123 (%) 58 (47)	N=724 (%) 314 (43)	N=959 (%) 430 (45)	0.21
Recreational (e.g. not being able to access recreational facilities like cinemas or restaurants, cancelled sports or cultural events)	N=112 (%) 66 (59)	N=123 (%) 61 (50)	N=740 (%) 306 (41)	N=975 (%) 433 (44)	0.001

### Communication, information and rumours

Table 4 shows the channels Thai residents rely on for information on COVID-19. The patterns of information acquisition on COVID-19 were similar across regions, except for the central region, where face-to-face meetings with healthcare professionals seemed to be limited. Traditional media, such as television, radio and newspapers, were the most common channels of communications. The government had made an effort to broadcast updates on the situations both on the national and global levels daily at midday, right from the beginning of the pandemic. A large proportion of Thai residents use mobile chat applications such as LINE, WhatsApp and Facebook messenger. This was reflected in the survey, with 88% of respondents indicating that they received information on social media or messenger apps. When it came to sharing information about COVID-19, 40% shared COVID-related information 1 to 3 times per month, less than 10% shared the information “very often”, whereas 16% reported that they did not share any information.

**Table 4.** Communication, information and rumours.

	Northern	Northeastern	Central	Southern	Eastern/Western	Total
	N=191 (%)	N=277 (%)	N=286 (%)	N=194 (%)	N=72 (%)	N=1,020
**How do/did you receive** **information about COVID-19?**						
Traditional media (TV, radio, newspapers)	188 (98)	272 (98)	275 (96)	160 (82)	71 (99)	966 (95)
Social media and messenger apps	169 (88)	248 (90)	266 (93)	163 (84)	54 (75)	900 (88)
Government/institution’s web page	158 (83)	239 (86)	226 (79)	130 (67)	65 (90)	818 (80)
Online (websites, email)	128 (67)	208 (75)	242 (85)	132 (68)	49 (68)	759 (74)
Face-to-face (e.g. doctors or health workers)	165 (86)	256 (92)	103 (36)	149 (77)	54 (75)	727 (71)
Print materials (leaflets, brochures)	121 (63)	182 (66)	95 (33)	78 (40)	52 (72)	528 (52)
WHO (World Health Organisation) web page	53 (28)	46 (17)	106 (37)	42 (22)	24 (33)	271 (27)
Scientific journals	50 (26)	39 (14)	112 (39)	38 (20)	20 (28)	259 (25)
University web pages	51 (27)	43 (16)	86 (30)	46 (24)	11 (15)	237 (23)
**How often do/did you share** **information about COVID-19** **in the last month?**						
Not at all	19 (10)	57 (21)	22 (8)	56 (29)	8 (11)	162 (16)
A little (1–3 per month)	91 (48)	118 (43)	122 (43)	59 (30)	23 (32)	413 (40)
Some (4–6 per month)	36 (19)	66 (24)	80 (28)	41 (21)	25 (35)	248 (24)
Often (7–9 per month)	28 (15)	20 (7)	35 (12)	22 (11)	10 (14)	115 (11)
Very often (10 or over per month)	17 (9)	16 (6)	27 (9)	16 (8)	6 (8)	82 (8)
**How would you prefer to** **receive information about** **COVID-19?**						
Media (TV, radio, newspapers)	182 (95)	264 (95)	265 (93)	161 (83)	67 (93)	939 (92)
Social media and messenger apps	166 (87)	240 (87)	256 (90)	166 (86)	55 (76)	883 (87)
Government/institution’s web page	170 (89)	238 (86)	248 (87)	144 (74)	66 (92)	866 (85)
Face-to-face (e.g. doctors or health workers)	168 (88)	264 (95)	165 (58)	164 (85)	58 (81)	819 (80)
Online (websites, email)	133 (70)	207 (75)	244 (85)	141 (73)	56 (78)	781 (77)
Print materials (leaflets, brochures)	123 (64)	195 (70)	130 (45)	103 (53)	48 (67)	599 (59)
WHO (World Health Organisation) web page	76 (40)	81 (29)	167 (58)	73 (38)	57 (79)	454 (45)
Scientific journals	72 (38)	69 (25)	152 (53)	75 (39)	53 (74)	421 (41)
University web page	74 (39)	72 (26)	125 (44)	80 (41)	48 (67)	399 (39)

People in all regions, except the Southern region, prefer to receive news from traditional media (92%), social media (87%) and government webpages (85%). More than 80% of the survey participants in all regions also prefer face-to-face communication, except for the central region where only 58% selected this option. University web pages, the WHO web page and scientific journals were not popular channels for COVID-19 information among Thai residents (less than 50%). When comparing the respondents’ received and preferred channels of information, traditional media, social media and government/institution’s web page remained the three most popular channels of information. There was a small increase in preferred channel of information received among face-to-face, print materials, online and government/institution’s web page compared with what have been received. There was a larger increase in preferred channel of information received among the academic sectors such as universities, WHO and scientific journals compared with how they did receive.

Table 5 summarised that many people had received unclear or conflicting information, especially about government support schemes (59%) and penalties for disobeying government restrictions (46%). These two topics were particularly highlighted in the Eastern and Western regions (82%). The percentage of unclear and conflicting information was highest among people from the Central region, i.e. above 50% in almost all topics except social distancing guidance (46%). The respondents felt that information related to the detection and control including social distancing guideline, dealing with symptoms, testing and risks from infection was the least conflicting information among all others, i.e. less than 40% in general. When asked about the ability to recognise fake news, around 5% admitted that they could not recognise fake news at all, while another 5% said they were very confident at recognising fake news. Almost 60% of respondents rated their level of understanding about COVID-19 as high, and 7% rated their level of understanding as ‘expert level’. Very few respondents (0.2%) indicated that they knew nothing about COVID-19 in our survey.

**Table 5.** Unclear or conflicting information about COVID-19 and level of understanding.

	Northern	Northeastern	Central	Southern	Eastern/ Western	Total	P-value
	N=191 (%)	N=277 (%)	N=286 (%)	N=194 (%)	N=72 (%)	N=1,020	
**Have you seen any unclear or conflicting information about COVID-19 in the last month?**							
Government support schemes (e.g. financial)	94 (49)	139 (50)	191 (67)	114 (59)	59 (82)	597 (59)	<0.001
Penalties if disobey restrictions	59 (31)	122 (44)	166 (58)	66 (34)	59 (82)	472 (46)	<0.001
Ways to avoid the infection	53 (28)	104 (38)	156 (55)	116 (60)	22 (31)	451 (44)	<0.001
Numbers of coronavirus cases/deaths related to COVID-19	57 (30)	107 (39)	156 (55)	89 (46)	26 (36)	435 (43)	<0.001
Symptoms	55 (29)	100 (36)	167 (58)	97 (50)	23 (32)	442 (43)	<0.001
Quarantine/isolation	57 (30)	97 (35)	149 (52)	82 (42)	29 (40)	414 (41)	<0.001
Travel restrictions (e.g. curfew, restricted hours of movement)	53 (28)	105 (38)	153 (53)	64 (33)	29 (40)	404 (40)	<0.001
Risks in case of infection	45 (24)	109 (39)	164 (57)	63 (32)	19 (26)	400 (39)	<0.001
Testing	67 (35)	98 (35)	153 (53)	60 (31)	19 (26)	397 (39)	<0.001
What to do in case of symptoms	49 (26)	95 (34)	149 (52)	74 (38)	19 (26)	386 (38)	<0.001
Social distancing guidance	53 (28)	96 (35)	132 (46)	76 (39)	22 (31)	379 (37)	<0.001
**How confident do you feel that you can recognize fake news about COVID-19?**							<0.001
Not at all	8 (4)	5 (2)	26 (9)	7 (4)	0 (0)	46 (5)	
A little	2 (1)	18 (6)	43 (15)	50 (26)	6 (8)	119 (12)	
Some	90 (47)	119 (43)	110 (38)	64 (33)	29 (40)	412 (40)	
A lot	82 (43)	113 (41)	96 (34)	66 (34)	35 (49)	392 (38)	
Very high/expert level	9 (5)	22 (8)	11 (4)	7 (4)	2 (3)	51 (5)	
**How would you rate your level of understanding of COVID-19?**							<0.001
Not at all	0 (0)	0 (0)	0 (0)	2 (1)	0 (0)	2 (0)	
A little	4 (2)	4 (1)	7 (2)	8 (4)	1 (1)	24 (2)	
Some	91 (48)	106 (38)	89 (31)	49 (25)	12 (17)	347 (34)	
A lot	88 (46)	140 (51)	175 (61)	123 (63)	54 (75)	580 (57)	
Very high/expert level	8 (4)	27 (10)	15 (5)	12 (6)	5 (7)	67 (7)	

### Coping and compliance with public health measures

From Table 6, over 92% of respondents reported that they changed their social behavior even before the implementation of government mandated strategies. A total of 94% reported socially distancing themselves from others, and 85% avoided physical contact with the older people and those with serious underlying conditions. A total 97% used personal protective equipment (e.g. masks) and 95% used sanitizer products even before government advice. Less than 50% moved from home to stay with parents/relatives. There was little variation in the reactions among the HCW and non-HCW group in coping and compliance indicators.

**Table 6.** Perceptions and compliance towards interventions among healthcare and general population.

	general HCW	local HCW	Non-HCW	Total
	N=55 (%)	N=125 (%)	N=764 (%)	N=1,020
Changing social behaviour before the implementation of government	50 (91)	125 (92)	764 (92)	939 (92)
**If ‘yes’ how social behaviour changed before the implementation of government restrictions?**				
Use of personal protection equipment (e.g. masks and gloves)	N=49 (%) 48 (98)	N=123 (%) 110 (89)	N=758 (%) 741 (98)	N=930 (%) 899 (97)
Use of sanitizer products and alcohol	N=48 (%) 47 (98)	N=121 (%) 109 (90)	N=737 (%) 709 (96)	N=906 (%) 865 (95)
No physical contact with anyone	N=49 (%) 48 (98)	N=124 (%) 117 (94)	N=758 (%) 711 (94)	N=931 (%) 876 (94)
No physical contact only with elderly and those with serious underlying conditions	N=48 (%) 45 (94)	N=122 (%) 102 (84)	N=715 (%) 606 (85)	N=885 (%) 753 (85)
Going out only for essential needs/work	N=49 (%) 45 (92)	N=121 (%) 104 (86)	N=753 (%) 705 (94)	N=923 (%) 854 (93)
Moving from home to stay with parents/relatives	N=48 (%) 24 (50)	N=122 (%) 55 (45)	N=715 (%) 356 (50)	N=885 (%) 435 (49)
**Maximum number of days you think you could cope without seeing anyone except the household members**				
1	2 (4)	4 (3)	62 (7)	68 (7)
2–7	24 (44)	29 (21)	231 (28)	284 (28)
8–14	22 (40)	33 (24)	255 (31)	310 (30)
15–21	2 (4)	15 (11)	75 (9)	92 (9)
22–28	2 (4)	8 (6)	48 (6)	58 (6)
more than 28 days	3 (5)	47 (35)	158 (19)	208 (20)
**Maximum number of days you think you could cope with not going out in public, assuming that you have** **sufficient supplies of food, medicines and other essential items**				
1	3 (5)	3 (2)	39 (5)	45 (4)
2–7	11 (20)	25 (18)	199 (24)	235 (23)
8–14	18 (33)	23 (17)	238 (29)	279 (27)
15–21	8 (15)	10 (7)	71 (9)	89 (9)
22–28	5 (9)	13 (10)	78 (9)	96 (9)
more than 28 days	10 (18)	62 (46)	204 (25)	276 (27)
**Maximum number of days you think you could cope with going out only for essential needs/work**				
1	2 (4)	3 (2)	37 (4)	42 (4)
2–7	18 (33)	24 (18)	200 (24)	242 (24)
8–14	19 (35)	29 (21)	238 (29)	286 (28)
15–21	9 (16)	15 (11)	72 (9)	96 (9)
22–28	5 (9)	8 (6)	74 (9)	87 (9)
more than 28 days	2 (4)	57 (42)	208 (25)	267 (26)

Around 35% reported that they could stay at home beyond 14 days without seeing family and friends outside their home, 45% would be able to manage beyond 14 days at home, assuming that they have sufficient supplies of food and essential items, and 44% could manage beyond 14 days when allowed to go out for essential items or work only. Local HCWs were most able to keep social distancing longer than other groups. Of local HCWs, 35% said that they would be willing to be home quarantined for 29 days or longer, while the responses from the general HCW and non-HCW were only 5 and 19%, respectively. The most acceptable length of time for self-quarantine among all groups was between 8 and 14 days in most circumstances.

From Table 7, on average 90% of the respondents across the regions answered that they would comply with government enforced or voluntary quarantine/isolation/social distancing. In all regions, over 90% (99% in some regions) of respondents agreed that the restrictions were necessary to control COVID-19. The majority seemed to be able to find ways to cope with the restrictions: More than 80% reported using social media for communication, engaging in self-care and exercising, and watching movies.

**Table 7.** Opinions and methods for coping with government enforced or voluntary quarantine/isolation/social distancing.

	Northern	Northeastern	Central	Southern	Eastern/Western	Total	p-value
	N=191 (%)	N=277 (%)	N=286 (%)	N=194 (%)	N=72 (%)	N=1,020	
**Statement**							
**I would comply with** **government enforced** **quarantine/isolation/social** **distancing**							<0.001
Strongly disagree	2 (1)	1 (0)	9 (3)	7 (4)	0 (0)	19 (2)	
Slightly disagree	1 (1)	3 (1)	6 (2)	7 (4)	0 (0)	17 (2)	
Neither agree nor disagree	0 (0)	7 (3)	17 (6)	43 (22)	5 (7)	72 (7)	
Slightly agree	70 (37)	136 (49)	117 (41)	97 (50)	46 (64)	466 (46)	
Strongly agree	118 (62)	130 (47)	137 (48)	40 (21)	21 (29)	446 (44)	
**I would enter voluntary** **quarantine/isolation/** **social distancing for social** **distancing**							<0.001
Strongly disagree	2 (1)	1 (0)	6 (2)	4 (2)	0 (0)	13 (1)	
Slightly disagree	0 (0)	2 (1)	1 (0)	4 (2)	0 (0)	7 (1)	
Neither agree nor disagree	6 (3)	14 (5)	32 (11)	20 (10)	4 (6)	76 (7)	
Slightly agree	93 (49)	161 (58)	126 (44)	117 (60)	45 (63)	542 (53)	
Strongly agree	90 (47)	99 (36)	121 (42)	49 (25)	23 (32)	382 (37)	
**Agreement with quarantine/** **isolation/social distancing** **(Yes/No)**							
I have been/would be able to participate in my work life.	189 (99)	272 (98)	278 (97)	188 (97)	70 (97)	997 (98)	0.63
I have been/would be able to participate in my private life.	189 (99)	272 (98)	279 (98)	183 (94)	70 (97)	993 (97)	0.048
It is a necessary strategy to help control COVID-19.	190 (99)	275 (99)	281 (98)	176 (91)	71 (99)	993 (97)	<0.001
**Methods for coping with** **quarantine/isolation/social** **distancing**							
Connecting with others (e.g. via phone, online or social media)	185 (97)	263 (95)	276 (97)	184 (95)	72 (100)	980 (96)	0.28
Self-care (e.g. exercise, healthy eating, meditation)	168 (88)	254 (92)	251 (88)	164 (85)	64 (89)	901 (88)	0.21
Engage in hobbies or learn new skills	156 (82)	254 (92)	251 (88)	119 (61)	55 (76)	835 (82)	<0.001
Watching movies or series (e.g. TV, Netflix)	159 (83)	227 (82)	250 (87)	123 (63)	59 (82)	818 (80)	<0.001
Finding alternative ways for things I enjoy doing (e.g. online classes or meetings)	148 (77)	212 (77)	245 (86)	118 (61)	60 (83)	783 (77)	<0.001

## Discussion

Our survey suggests that good understanding of disease, interventions and the positive perceptions towards government interventions may have contributed to the success of the disease control we see for Thailand during the first outbreak of COVID-19. Positive perception of and level of tolerance for the enforced interventions were high. In general, respondents tended to cope well with the government implementation of control interventions including quarantine/isolation/social distancing, and found activities such as being connected using social media and self-caring exercises to be necessary.

A large proportion of the population in Bangkok and other cities have already been wearing masks to protect themselves from PM2.5 air pollutants since the end of 2019 ^11^. The biggest impacts felt by Thai residents during the first wave of the pandemic were loss of income, concerns of physical health and increased caring responsibilities. This particularly applied to those who live with extended family, which is common in the country. People who live alone tended to be concerned about mental health and their social life.

The impacts on the Thai economy have been significant, because the country has been an important trade and tourism hub. The COVID-19 pandemic hit a significant number of local small and medium-sized enterprises (SMEs) hard, with these having generated 43% of Thailand’s GDP in 2019. Prior to COVID-19, the unemployment rate was approximately 1% in 2019 and now a survey suggested that the figure could reach 10% ^12^. A decline of the Thai economy by 5% in 2020 is projected to be the sharpest in the East Asia and Pacific Regions ^13^. The tourism sector in the country accounts for approximately 15% of the GDP. China is Thailand’s biggest source of foreign tourists, accounting for 28 percent of the 39.8 million visitors last year ^14^. This was reflected in our survey results, as a very high number of respondents reported loss of earnings.

Good communication during the pandemic is essential to keep people informed on the national and global situation, and to remind people to comply with the government strategies. A previous study reported a significant increase in the level of anxiety among Thai residents, especially among the younger generation ^15^ and healthcare workers ^16^. Anxiety was said to be motivating both desirable and undesirable behaviours during pandemic outbreaks. Effective and targeted communication by trusted sources is needed to motivate preventive actions ^15^. The daily briefings of Thailand’s Centre for COVID-19 Situation Administration (CCSA) on a national and global scale have been broadcast early since January on the Government’s website and others. Public messaging and social media should support public health responses by teaming up with the Government in providing consistent, simple and clear messaging, since either positive or negative messages can influence the public ^17^.

This study showed a high level of cooperation by people to the government-enforced or voluntarily controls such as quarantine, isolation and social distancing regardless of geographical regions. There was a high level of cooperation by people to the government-enforced or voluntarily controls such as quarantine, isolation and social distancing regardless of geographical regions or being HCW, almost 18% of all respondents. In addition, local HCWs indicated the highest tolerance for longer self-quarantine in all circumstances, possibly due to the nature of their work of non-business type with fixed income. Further studies on perspectives of healthcare workers would be useful for confirming this. Similar level of coping and compliance during the pandemic among people indicated that the population in general had received good information about COVID-19, government strategies and good health practices.

There has been a lot of public health messaging on face mask wearing and hand hygiene on Thai mass media channels and healthcare networks. A recent study among COVID-19 patients and their contacts in Thailand showed that wearing masks, washing hands and social distancing were strongly associated with lower risk of COVID-19 infections ^18^. The evidence that mask wearing can help protect people from the infection has become more obvious, and this has resulted in updates of international guidelines related to mask wearing ^19, 20^. The level of compliance of mask wearing is high in public areas. In addition, people had a sense of social responsibility to help the country get through this crisis by not letting their guard down, keeping social distance, wearing a face mask/cloth mask, and frequently washing hands ^21^.

This study is one of a few studies that assessed the impact and perceptions of COVID-19 and its public health measures on the general population in Thailand. Others focused on healthcare worker or on mental health issues ^16, 22^. Our online survey provided real-time responses for monitoring the public perception over time while the situation and policy decisions were very dynamic.

However, one limitation of online survey was limited access to smartphone and digital technology in some settings such as rural areas of Thailand. This was unavoidable as we could not have feasibly conducted a paper-based survey during a pandemic. Our recruitment strategy was non-purposive thus the survey cohort are not nationally representative. We have tried to minimize this bias by using a professional polling service with a wide network of contacts. The findings from this study can be incorporated into government planning to control the pandemic and improve communications with the general public in the future.

## Data availability

### Underlying data

Zenodo: Dataset for: Perspectives on public health interventions in the management of the COVID-19 pandemic in Thailand, http://doi.org/10.5281/zenodo.4030513 ^10^.

This project contains the following underlying data:

- Dataset - Online survey Social, ethical and behavioural aspects of COVID-19 (SEBCOV)_Thailand.xlsx (Survey responses)

### Extended data

Zenodo: Online survey questions: Social, ethical and behavioural aspects of COVID-19, http://doi.org/10.5281/zenodo.4049821 ^6^.

This project contains the following extended data:

- SEBCOV_Survey_AllVersions_V1.0_24Apr2020.pdf (Survey questions)

Data are available under the terms of the Creative Commons Attribution 4.0 International license (CC-BY 4.0).
