# Supplementary material for: Perspectives on public health interventions in the management of the COVID-19 pandemic in Thailand
Source: Wellcome Open Res. 2026 Oct 15;5:245. Originally published 2020 Oct 19. [Version 4] doi: 10.12688/wellcomeopenres.16293.4 (PMC8283548; doi:10.12688/wellcomeopenres.16293.4)
Supplement: Peer review history

# Reviewer report: Reviewer response for version 3

**DOI:** 10.21956/wellcomeopenres.18846.r44984

**Published:** 2021-07-15

**Authors:** Robin Goodwin, Juthatip Wiwattanapantuwong

**Affiliations:** 1 Department of Psychology, University of Warwick, Coventry, UK; 2 Department of Psychology, Chulalongkorn University, Bangkok, Thailand

Thank you the authors for the revision. We are happy with this version of the manuscript.

Is the work clearly and accurately presented and does it cite the current literature?

Yes

If applicable, is the statistical analysis and its interpretation appropriate?

Partly

Are all the source data underlying the results available to ensure full reproducibility?

Partly

Is the study design appropriate and is the work technically sound?

Yes

Are the conclusions drawn adequately supported by the results?

Yes

Are sufficient details of methods and analysis provided to allow replication by others?

Partly

Reviewer Expertise:

NA

We confirm that we have read this submission and believe that we have an appropriate level of expertise to confirm that it is of an acceptable scientific standard.

# Reviewer report: Reviewer response for version 3

**DOI:** 10.21956/wellcomeopenres.18846.r44983

**Published:** 2021-07-15

**Authors:** Pilasinee Wongnuch

**Affiliations:** 1 School of Health Sciences, Mae Fah Luang University, Chiang Rai, Thailand

Thank you for the opportunity to review this interesting paper on this really important topic. This revision has better balance and response to all reviewer comments, I have no further comments to make.

 I look forward to hearing this paper has been indexed, the content including the recommendations will reach those who need it.

Is the work clearly and accurately presented and does it cite the current literature?

Yes

If applicable, is the statistical analysis and its interpretation appropriate?

Yes

Are all the source data underlying the results available to ensure full reproducibility?

Yes

Is the study design appropriate and is the work technically sound?

Partly

Are the conclusions drawn adequately supported by the results?

Yes

Are sufficient details of methods and analysis provided to allow replication by others?

Yes

Reviewer Expertise:

Medical anthropology

I confirm that I have read this submission and believe that I have an appropriate level of expertise to confirm that it is of an acceptable scientific standard.

# Reviewer report: Reviewer response for version 2

**DOI:** 10.21956/wellcomeopenres.18119.r44572

**Published:** 2021-06-29

**Authors:** Pilasinee Wongnuch

**Affiliations:** 1 School of Health Sciences, Mae Fah Luang University, Chiang Rai, Thailand

This study highlights the impacts and perceptions of COVID-19 and its public health measures on the general population in Thailand which collecting the data through online survey. 1,020 participants from all regions were recruited. The results have shown the economic impacts of COVID-19 and related government interventions especially trade and tourism hub. While the cooperation by people to the government-enforced or voluntarily controls was a high level. It is indeed a timely and interesting piece of research. May I please make the following suggestions to your manuscript? Thank you.

 1. The Method section:

**Study participants and recruitment**

 In case of sampling technique, if you could please consider revising this section with probability method by using a list-based sampling frame  which conducted via e-mail addresses and social media accounts, including non-probability sampling methods with unrestricted self-selected surveys by posting invitations to participate via recruitment posters. (I highly recommended the article of Ronald D. Fricker. (2008). Sampling Methods for Online Surveys, https://study.sagepub.com/sites/default/files/Fricker.pdf). Perhaps you would give some citations to support that method. This would add to the strength of the academic foundation of your manuscript.

**Sample size**

 1,020 participants provide enough support for your finding credibility, if you could please consider revising this section to focus more on the sample size calculation by using the prevalence of preparedness or health behavior for COVID-19 prevention from a previous study in a familiar population. I think it would support the academic merit for your paper.

Is the work clearly and accurately presented and does it cite the current literature?

Yes

If applicable, is the statistical analysis and its interpretation appropriate?

Yes

Are all the source data underlying the results available to ensure full reproducibility?

Yes

Is the study design appropriate and is the work technically sound?

Partly

Are the conclusions drawn adequately supported by the results?

Yes

Are sufficient details of methods and analysis provided to allow replication by others?

Yes

Reviewer Expertise:

Medical anthropology

I confirm that I have read this submission and believe that I have an appropriate level of expertise to confirm that it is of an acceptable scientific standard, however I have significant reservations, as outlined above.

# Response

**Published:** 2021-07-10

**Authors:** Wirichada Pan-ngum

**Affiliations:** Mahidol Oxford Tropical Medicine Research Unit, Thailand

10 ^th^ July 2021

 Dear Dr. Pilasinee Wongnuch and Editor

 Thank you for the constructive comments and suggestions to our work. We have responded point-by-point below.

 Yours sincerely,

 Wirichada Pan-ngum (corresponding author)

 #################################################################

 This study highlights the impacts and perceptions of COVID-19 and its public health measures on the general population in Thailand which collecting the data through online survey. 1,020 participants from all regions were recruited. The results have shown the economic impacts of COVID-19 and related government interventions especially trade and tourism hub. While the cooperation by people to the government-enforced or voluntarily controls was a high level. It is indeed a timely and interesting piece of research. May I please make the following suggestions to your manuscript? Thank you.

 1. The Method section:

 Study participants and recruitment

 In case of sampling technique, if you could please consider revising this section with probability method by using a list-based sampling frame which conducted via e-mail addresses and social media accounts, including non-probability sampling methods with unrestricted self-selected surveys by posting invitations to participate via recruitment posters. (I highly recommended the article of Ronald D. Fricker. (2008). Sampling Methods for Online Surveys, https://study.sagepub.com/sites/default/files/Fricker.pdf). Perhaps you would give some citations to support that method. This would add to the strength of the academic foundation of your manuscript.

*We agree with the suggestion and have edited this part to include the suggested text and reference.*

 Sample size

 1,020 participants provide enough support for your finding credibility, if you could please consider revising this section to focus more on the sample size calculation by using the prevalence of preparedness or health behavior for COVID-19 prevention from a previous study in a familiar population. I think it would support the academic merit for your paper.

*We have followed the advice and re-written the section on sample size to include some references and formulae.*

# Reviewer report: Reviewer response for version 2

**DOI:** 10.21956/wellcomeopenres.18119.r41705

**Published:** 2020-12-08

**Authors:** Robin Goodwin, Juthatip Wiwattanapantuwong

**Affiliations:** 1 Department of Psychology, University of Warwick, Coventry, UK; 2 Department of Psychology, Chulalongkorn University, Bangkok, Thailand

We have now looked at the revised paper and are very grateful for the amendments made by the author. We thank them for their hard work and pleased to provide our approval of the revision.

Is the work clearly and accurately presented and does it cite the current literature?

Yes

If applicable, is the statistical analysis and its interpretation appropriate?

Partly

Are all the source data underlying the results available to ensure full reproducibility?

Partly

Is the study design appropriate and is the work technically sound?

Yes

Are the conclusions drawn adequately supported by the results?

Yes

Are sufficient details of methods and analysis provided to allow replication by others?

Partly

Reviewer Expertise:

NA

We confirm that we have read this submission and believe that we have an appropriate level of expertise to confirm that it is of an acceptable scientific standard.

# Reviewer report: Reviewer response for version 1

**DOI:** 10.21956/wellcomeopenres.17903.r41002

**Published:** 2020-10-23

**Authors:** Robin Goodwin, Juthatip Wiwattanapantuwong

**Affiliations:** 1 Department of Psychology, Chulalongkorn University, Bangkok, Thailand; 2 Department of Psychology, University of Warwick, Coventry, UK

Studying behavioural adaptation to COVID-19 and subsequent preventive behaviour is an important topic for research, and as the first country to experience cases of this novel coronavirus outside of Thailand this is an important nation for analysis. Bangkok is the world's most visited city and therefore the implications of potential spread were indeed far reaching. As of late October 2020 Thailand has dealt very well with the outbreak, with relatively low numbers of cases and small rates of morbidity, thus helping provide a possible model for rapid and successful intervention early on in an outbreak.

 The submitted manuscript is well written and structured, with generally clear information about the process of participant recruitment and the data analysis. The authors conducted an online survey in Thailand during May 2020 using snowball sampling and a reasonably-large sample (N=1020). (Purely by coincidence our team at Chulalongkorn/Warwick Universities were conducting a similar sized nationally representative face-to-face survey at the very same time, funded by a small GCRF grant awarded to Warwick and working with a national Thai survey company). The team at the Mahidol-Oxford Tropical Medicine Research Unit piloted their questionnaire in five different countries. Despite the online method the team managed to obtain a reasonable number of older respondents, often difficult to gather through the methods they employed. 

 Respondents' greatest concern was over the economic impact of the coronavirus, no doubt reflecting the profound impact of the virus on the tourist industry as well as other sectors. Respondents had a clear preference for information on traditional media (something we also found in our cotemporaneous national survey); they also reported high levels of preference for social media, although this may partly reflect the online nature of the study. The respondents report high levels of anticipatory compliance (even before government enforcement). This may reflect the considerable number of community activities that actively encouraged this, even within more remote settings. The authors rightly conclude that carefully targeted information is vital; to be most efficacious this is likely to stress both personal health and economic benefits of preventive action plus the wider societal gains from such action.

 The work is interesting and important, but we have some suggestions for minor improvements that could further strengthen the paper. 

1. It would be useful to provide detail of the number of respondents per region (e.g. were most in Bangkok?). This should be in the main text rather than the underlying datasets.
2. More information on the scales used would be useful (e.g. are they allowing multiple answers, what were scale end points)?
3. We note that, on p.4, the authors discuss occurrence of fake news items and self-reported understanding of COVID-19 but we could not see these discussed in any further detail later in the article.
4. For Table 2 'inconvenience caused by COVID-19' , and Table 5 'how social behaviour changed before the implementation of government restrictions?', please mention the reason why the N for each column differed from others in the same column.  
5. Table 3, please confirm that when the authors mention examples in the categories; such as, financial (e.g. loss of income, loss of job), were these asked as separate items?
6. For all tables, we suggest the authors arrange items in the same topic by frequency order.
7. When the authors mentioned % of the way participants 'receive' and 'prefer to receive' the information, It would be helpful if the authors consider the ways there are differences in scores between those these two. While 'traditional media' are mostly mentioned for both questions, they preferred to receive them 'less' than they already received (not clear if this is statistically significant or not). In contrast, the number of participants who preferred to receive information about COVID-19 via the university website, WHO, and scientific journals are clearly higher than those who mentioned they received such information. 
8. Having 17.65% of participants as health care workers, and participants from every area in Thailand, it would be helpful if the authors could provide more implications of their findings related to these occupational/regional variables. 

Is the work clearly and accurately presented and does it cite the current literature?

Yes

If applicable, is the statistical analysis and its interpretation appropriate?

Partly

Are all the source data underlying the results available to ensure full reproducibility?

Partly

Is the study design appropriate and is the work technically sound?

Yes

Are the conclusions drawn adequately supported by the results?

Yes

Are sufficient details of methods and analysis provided to allow replication by others?

Partly

Reviewer Expertise:

Psychology (social psychology, health psychology).

We confirm that we have read this submission and believe that we have an appropriate level of expertise to confirm that it is of an acceptable scientific standard, however we have significant reservations, as outlined above.

# Response

**Published:** 2020-11-17

**Authors:** Wirichada Pan-ngum

**Affiliations:** Mahidol Oxford Tropical Medicine Research Unit, Thailand

Dear Dr. Juthatip Wiwattanapantuwong, Prof. Robin Goodwin, and Editor

 Thank you for the constructive comments, suggestions and critiques. We have responded point-by-point below in *ltalic* and some points involved revising the manuscript which we are submitting.

 Yours sincerely,

 Wirichada Pan-ngum (corresponding author)

 #################################################

 Studying behavioural adaptation to COVID-19 and subsequent preventive behaviour is an important topic for research, and as the first country to experience cases of this novel coronavirus outside of Thailand this is an important nation for analysis. Bangkok is the world's most visited city and therefore the implications of potential spread were indeed far reaching. As of late October 2020 Thailand has dealt very well with the outbreak, with relatively low numbers of cases and small rates of morbidity, thus helping provide a possible model for rapid and successful intervention early on in an outbreak.

*Thank you for the great introduction and couldn’t agree more.*

 The submitted manuscript is well written and structured, with generally clear information about the process of participant recruitment and the data analysis. The authors conducted an online survey in Thailand during May 2020 using snowball sampling and a reasonably-large sample (N=1020). (Purely by coincidence our team at Chulalongkorn/Warwick Universities were conducting a similar sized nationally representative face-to-face survey at the very same time, funded by a small GCRF grant awarded to Warwick and working with a national Thai survey company). The team at the Mahidol-Oxford Tropical Medicine Research Unit piloted their questionnaire in five different countries. Despite the online method the team managed to obtain a reasonable number of older respondents, often difficult to gather through the methods they employed.

*Despite limited use of smartphones and computers among the elderly in general, we were able to recruit 6% (62/1020) from population aged 65 and more through the online survey. There was a similar proportion of population from this group from different regions of Thailand.*

 Respondents' greatest concern was over the economic impact of the coronavirus, no doubt reflecting the profound impact of the virus on the tourist industry as well as other sectors. Respondents had a clear preference for information on traditional media (something we also found in our cotemporaneous national survey); they also reported high levels of preference for social media, although this may partly reflect the online nature of the study. The respondents report high levels of anticipatory compliance (even before government enforcement). This may reflect the considerable number of community activities that actively encouraged this, even within more remote settings. The authors rightly conclude that carefully targeted information is vital; to be most efficacious this is likely to stress both personal health and economic benefits of preventive action plus the wider societal gains from such action.

 The work is interesting and important, but we have some suggestions for minor improvements that could further strengthen the paper.

 It would be useful to provide detail of the number of respondents per region (e.g. were most in Bangkok?). This should be in the main text rather than the underlying datasets.

*The number of respondents per region was provided in the Table 1. We have added to the main text the description to say “The breakdown of respondents by region is as follows: Central (28%), Northeastern (27%), Southern (19%), Northern (19%) and Eastern/Western (7%).”*

 More information on the scales used would be useful (e.g. are they allowing multiple answers, what were scale end points)?

*We have added to the method section in the study design “The 5-point Likert-scale, ranged from strongly agree to strongly disagree, was used for opinions on statements reflecting the opinions on the government restrictions”.*

 We note that, on p.4, the authors discuss occurrence of fake news items and self-reported understanding of COVID-19 but we could not see these discussed in any further detail later in the article.

*We have followed the advice from the reviewers and added an extra table (Table 5) to discuss the possible topics of fake news, level of confidence among the respondents in recognizing this, and self-reported understanding of COVID-19.*

*Extra text to describe the conflicting information and confidence about recognition of fake news goes in the end of section 3.3*

*“In summary, many people had received unclear or conflicting information, especially about government support schemes (59%) and penalties for disobeying government restrictions (46%). These two topics were particularly highlighted in the Eastern and Western regions (82%). The percentage of unclear and conflicting information was highest among people from the Central region, i.e. above 50% in almost all topics except social distancing guidance (46%).  The respondents felt that information related to the detection and control including social distancing guideline, dealing with symptoms, testing and risks from infection was the least conflicting information among all others, i.e. less than 40% in general. When asked about the ability to recognise fake news, around 5% admitted that they could not recognise fake news at all, while another 5% said they were very confident at recognising fake news. Almost 60% of respondents rated their level of understanding about COVID-19 as high, and 7% rated their level of understanding as ‘expert level’. Very few respondents (0.2%) indicated that they knew nothing about COVID-19 in our survey. “*

 For Table 2 'inconvenience caused by COVID-19', and Table 5 'how social behaviour changed before the implementation of government restrictions?', please mention the reason why the N for each column differed from others in the same column. 

*In Table 2, 3 and 5 we specified the number of those who responded to each question and used this number as the denominator instead of the total number of respondents in the main column headings. Basically, the missing responses were not taken into the calculations.*

 Table 3, please confirm that when the authors mention examples in the categories; such as, financial (e.g. loss of income, loss of job), were these asked as separate items?

*In Table 3, ’loss of income’ and ‘loss of job’ were combined under one question about ‘financial concerns’.*

 For all tables, we suggest the authors arrange items in the same topic by frequency order.

*We have followed the reviewer’s suggestion and make appropriate corrections in*

*Table 2: **Any inconvenience caused by COVID-19***

*Table 3: **Concerns when no physical contact/not allowed to go out/allowed to go out only for essential needs***

*Table 4: **How do/did you receive information about COVID-19?***

*And **How would you prefer to receive information about COVID-19?***

*New Table 5 : **Have you seen** **any unclear or conflicting information about COVID-19  in the last month?***

*New Table 6: **If ‘yes’ how social behaviour changed before the implementation of government restrictions?***

*New Table 7: **Methods for coping with quarantine/isolation/social distancing***

 When the authors mentioned % of the way participants 'receive' and 'prefer to receive' the information, It would be helpful if the authors consider the ways there are differences in scores between those these two. While 'traditional media' are mostly mentioned for both questions, they preferred to receive them 'less' than they already received (not clear if this is statistically significant or not). In contrast, the number of participants who preferred to receive information about COVID-19 via the university website, WHO, and scientific journals are clearly higher than those who mentioned they received such information.

*We added to the text*

*“In Table 4 when comparing the respondents’ received and preferred channels of information, traditional media, social media and government/institution’s web page remained the three most popular channels of information. There was a small increase in preferred channel of information received among face-to-face, print materials, online and government/institution’s web page compared with what have been received. There was a larger increase in preferred channel of information received among the academic sectors such as universities, WHO and scientific journals compared with how they did receive. “*

 Having 17.65% of participants as health care workers, and participants from every area in Thailand, it would be helpful if the authors could provide more implications of their findings related to these occupational/regional variables.

*In our study, around 18% of the respondents were in healthcare sectors, both fulltime (5%) and health volunteer staff or local HCW (13%). The fact that similar level of coping and compliance during the pandemic was obtained between HCW and non-HCW, indicated that the Thai population in general had received good information about COVID-19, government strategies and good health practices. Local HCWs indicated the highest tolerance for longer self-quarantine in all circumstances, possibly due to the nature of their work of non-business type with fixed income. Further studies on perspectives of healthcare workers would be useful for confirming this.*

*We have adjusted the discussion to include this point in the revised version.*

*“This study showed a high level of cooperation by people to the government-enforced or voluntarily controls such as quarantine, isolation and social distancing regardless of geographical regions or being HCW, almost 18% of all respondents. In addition, local HCWs indicated the highest tolerance for longer self-quarantine in all circumstances, possibly due to the nature of their work of non-business type with fixed income. Further studies on perspectives of healthcare workers would be useful for confirming this.*

*Similar level of coping and compliance during the pandemic among people indicated that the the population in general had received good information about COVID-19, government strategies and good health practices.”*
